# Phytochemical Profiling, Antioxidant and Tyrosinase Regulatory Activities of Extracts from Herb, Leaf and In Vitro Culture of *Achillea millefolium* (Yarrow)

**DOI:** 10.3390/molecules28124791

**Published:** 2023-06-15

**Authors:** Karolina Czech, Katarzyna Gaweł-Bęben, Agnieszka Szopa, Wirginia Kukula-Koch, Thomas Jakschitz, Günther Bonn, Shah Hussain, Paweł Kubica, Halina Ekiert, Kazimierz Głowniak

**Affiliations:** 1 Department of Cosmetology, University of Information Technology and Management in Rzeszów, Sucharskiego 2, 35-225 Rzeszow, Poland; kczech@wsiz.edu.pl (K.C.); kglowniak@wsiz.edu.pl (K.G.); 2 Chair and Department of Pharmaceutical Botany, Medical College, Jagiellonian University, Medyczna 9, 30-688 Cracow, Poland; a.szopa@uj.edu.pl (A.S.); p.kubica@uj.edu.pl (P.K.); halina.ekiert@uj.edu.pl (H.E.); 3 Department of Pharmacognosy with Medicinal Plants Garden, Medical University of Lublin, Chodźki 1, 20-093 Lublin, Poland; 4 Austrian Drug Screening Institute GmbH, Innrain 66a, 6020 Innsbruck, Austria; thomas.jakschitz@adsi.ac.at (T.J.); guenther.bonn@adsi.ac.at (G.B.); shah.hussain@adsi.ac.at (S.H.)

**Keywords:** yarrow herb, yarrow leaves, shoot culture, UHPLC-hr-qTOF/MS, tissue culture, antioxidant activity, tyrosinase inhibitory activity, plant biotechnology

## Abstract

*Achillea millefolium* L. is one of the most known medicinal plants with a broad spectrum of applications in the treatment of inflammation, pain, microbial infections and gastrointestinal disorders. In recent years, the extracts from *A. millefolium* have also been applied in cosmetics with cleansing, moisturizing, shooting, conditioning and skin-lightening properties. The growing demand for naturally derived active substances, worsening environmental pollution and excessive use of natural resources are causing increased interest in the development of alternative methods for the production of plant-based ingredients. In vitro plant cultures are an eco-friendly tool for continuous production of desired plant metabolites, with increasing applicability in cosmetics and dietary supplements. The purpose of the study was to compare phytochemical composition and antioxidant and tyrosinase inhibitory properties of aqueous and hydroethanolic extracts from *A. millefolium* obtained from field conditions (AmL and AmH extracts) and in vitro cultures (AmIV extracts). In vitro microshoot cultures of *A. millefolium* were obtained directly from seeds and harvested following 3 weeks of culture. Extracts prepared in water, 50% ethanol and 96% ethanol were compared for the total polyphenolic content, phytochemical content using the ultra-high-performance liquid chromatography–quadrupole time-of-flight mass spectrometry (UHPLC-hr-qTOF/MS), antioxidant activity by DPPH scavenging assay and the influence on the activity of mushroom and murine tyrosinases. The phytochemical content of AmIV extracts was significantly different from AmL and AmH extracts. Most of the polyphenolic compounds identified in AmL and AmH extracts were present in AmIV extracts only in trace amounts and the major constituents presented in AmIV extracts were fatty acids. The total content of polyphenols in AmIV exceeded 0.25 mg GAE/g of dried extract, whereas AmL and AmH extracts contained from 0.46 ± 0.01 to 2.63 ± 0.11 mg GAE/g of dried extract, depending on the solvent used. The low content of polyphenols was most likely responsible for the low antioxidant activity of AmIV extracts (IC_50_ values in DPPH scavenging assay >400 µg/mL) and the lack of tyrosinase inhibitory properties. AmIV extracts increased the activity of mushroom tyrosinase and tyrosinase present in B16F10 murine melanoma cells, whereas AmL and AmH extracts showed significant inhibitory potential. The presented data indicated that microshoot cultures of *A. millefolium* require further experimental research before they can be implemented as a valuable raw material for the cosmetics industry.

## 1. Introduction

*Achillea millefolium* L. (yarrow, family Asteraceae) is one of the most studied medicinal plants worldwide, known for a long history of application in traditional and modern medicines. The broad spectrum of the beneficial properties of *A. millefolium* includes treatment of inflammation, pain, gastrointestinal disorders and anti-bacterial, anti-plasmodial and anti-ulcer activities [1]. Extracts obtained from this plant are also broadly used for skin applications due to their anti-inflammatory, skin-lightening, wound-healing, rejuvenating and anti-microbial properties [2]. In cosmetics, the extracts from herbs, leaves or flowers of *A. millefolium* are recommended as cleansing, moisturizing, shooting, conditioning, masking and refreshing ingredients [3]. Extracts obtained from herbs, leaves and flowers of *A. millefolium* are also rich in natural antioxidants—valuable active ingredients of anti-aging and skin-protecting cosmetics [2]. Recent studies also showed the skin-lightening potential of *A. millefolium* hydroalcoholic and hydroglycolic extracts [2,4,5].

The broad spectrum of biological activities results from the exceptionally rich phytochemical composition of *A. millefolium*. The most common compounds include flavonoids (glucosilated and nonglucosilated), phenolic acids (mostly caffeic, cinnamic and benzoic acid derivatives), terpenes (guaianolides, diterpens, sesquiterpenes and their oxygenated forms), phytosterols, organic acids, fatty acids and alcohols [6]. Flavonoids and phenolic acids (especially caffeoylquinic acid derivatives) are responsible for significant antioxidant properties of *Achillea* spp. extracts [7,8] and phytosterols are known for their skin calming potential [9,10]. The presence of guaianolides, especially α-peroxyachifolid, might be responsible for the development of allergic contact dermatitis upon topical application of *A. millefolium* extracts. The concentration of this compound varies in fresh plant material from up to 0.6% in blossoms to 0.05% in leaves and decreases during the drying or processing of plant material due to the degradation of compounds [11]. The other compounds of concern in respect of the safety of *A. millefolium* extracts are linalool, thujone, quercetin and hydroquinone, but their content in the plant and in the extracts is very low, not exceeding the safe dose. Therefore, based on the current scientific data, *A. millefolium* extracts are considered safe to use as cosmetic ingredients [12].

Today, when choosing food and cosmetics, most consumers prefer to use natural products with a low ecological footprint [13]. Due to the growing demand for active substances and extracts of natural origin, as well as increasing environmental pollution and excessive use of natural resources, the usage of alternative sources of biologically active natural ingredients are sought. In vitro cultures of plant cells, tissues and organs under a controlled environment are an important tool for the continuous and eco-friendly production of plant metabolites [14]. Moreover, extracts of plant in vitro cultures are becoming the innovative ingredient of dietary supplements and especially cosmetics due to the fact that they are a unique blend of secondary and primary metabolites naturally occurring in plant cells [15,16]. However, for each plant species, the conditions of in vitro cultures must be carefully optimized in order to obtain satisfactory levels of bioactive metabolites and possibly reduce amounts of potentially harmful compounds [17]. In vitro cultures of *A. millefolium* have not been an object of such extensive research. Alvarenga et al., investigated the content of volatile compounds in *A. millefolium* in vitro cultures obtained from rhizomes exposed to different light spectra [18]. Figueiredo et al., established the conditions for *A. millefolium* cell suspension in vitro cultures and compared the phytochemical composition of the essential oil obtained from this culture with the essential oil produced from the plant grown in wild. The composition of the essential oil from the cell suspension cultures differed markedly from that of the wild grown plant. The monoterpene fraction was reduced and the major compounds of the essential oil were eugenol and demethoxyencecalin, whereas 1,8-cineole and sabinene were the most abundant constituents of essential oils obtained from the wild-growing plant [19]. These data suggest that the phytochemical composition of *A. millefolium* grown in vitro significantly varies from the wild-growing plant and requires further studies in order to evaluate their utilization for pharmaceutical and cosmetic applications.

The aim of this study was to establish the conditions of the in vitro microshoot cultures initiated from the seeds of *A. millefolium* and to compare the phytochemical content, antioxidant and tyrosinase inhibitory activities of aqueous, hydroethanolic (50% EtOH) and ethanolic (96% EtOH) extracts of *A. millefolium* herbs (AmH) and leaves (AmL) harvested from the field or obtained from an innovative in vitro culture (AmIV).

## 2. Results and Discussion

### 2.1. Achillea millefolium Microshoot Cultures

**Figure 1:**
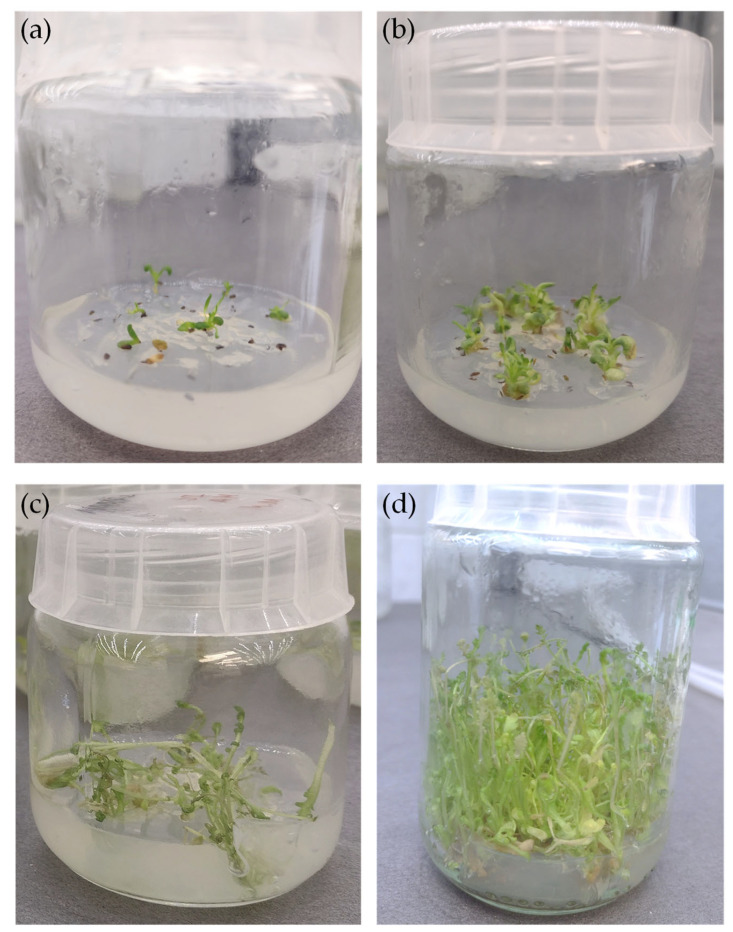
In vitro cultures of *Achillea millefolium*; (**a**) establishment of in vitro culture from seeds, (**b**) in vitro culture seedlings obtained after 1 week; (**c**) microshoot cultures obtained after a 2-week growth period; (**d**) microshoot cultures after a 3-week growth period.

The *A. millefolium* microshoots were established from the seeds and were cultivated on Murashige and Skoog (MS) medium supplemented with 1 mg/L BA, 0.5 mg/L NAA and 0.2 mg/L GA_3_ (Figure 1). After a 3-week growth period, the cultures were characterized by a numerous microshoots and the light green color of the shoots and leaves (Figure 1d). The average growth index (Gi) was relatively high, equal to 87.81 ± 0.69%.

Growth conditions for the established *A. millefolium* microshoot cultures were estimated based on the available reports investigating the conditions of the optimal in vitro growth and micropropagation of *Achillea* ssp. and other plant species. Recent studies on the accumulation and production of valuable secondary metabolites of different plant species were based on the examination of various factors that may affect these parameters. For example, in the work by Szopa et al., it was examined that the different ratio of auxins/cytokines significantly affects not only the growth of biomass but also the content and composition of active compounds [20]. Turker et al., described an in vitro plant regeneration protocol for shoot-tip or root explants of *A. millefolium*, excised from in vitro-grown seedlings and cultures grown on MS medium with different concentrations and combinations of various plant growth regulators, including BA, NAA, GA_3_, IBA (indole-3-butyric acid), IAA (indole-3-acitic acid) and KIN (kinetin). All of the tested regulations significantly influenced the size and number of explants in the culture [21]. This study, however, did not include the phytochemical analysis of obtained plant material. Grigoriadou et al., investigated the influence of NAA, BA and IBA on the growth and rooting of shoot-tip cultures of *A. occulata*, showing that the combination of BA and IBA increased the proliferation of shoots whereas the treatment with IBA and NAA increased rooting [22].

Other works compared the effect of light intensity and wavelength on the cultivation of plant cultures and on the obtained extraction material [23,24]. Alvarenga et al., examined the influence of the light emitting diode (LED) lamps in the blue, red, green and white wavelengths and various light intensities on the growth and volatiles production by *A. millefolium* in vitro cultures established from terminal segments of rhizomes obtained from greenhouse cultivation. The color of light as well as the intensity significantly changed the content of volatiles, chlorophyll and carotenoid profiles present in plant material, indicating that light is also an important factor regulating the biosynthesis of active compounds by *A. millefolium* [18]. 

### 2.2. Comparative Study on Total Polyphenols and Antioxidant Activity

Aqueous (H_2_O), hydroethanolic (50% EtOH) and ethanolic (96% EtOH) extracts from *A. millefolium* in vitro culture (AmIV) were compared for the total polyphenolic content and DPPH scavenging activity with the extracts obtained from herbs (AmH) and leaves (AmL) of this species harvested from nature (Table 1).

For all analyzed plant samples, 50% EtOH extracts contained the highest amounts of polyphenolic compounds and showed the highest antioxidant potential in the DPPH radical scavenging assay. The extracts obtained from AmH and AmL contained about 7–10 times higher amounts of polyphenols than AmIV extracts prepared using the same solvent. Low content of polyphenols was also correlated with almost undetectable DPPH scavenging activity of AmIV extracts.

The content of secondary metabolites such as polyphenolic compounds is a very important factor influencing the multitude of medicinal properties of plant material. Polyphenolic compounds are also important constituents of *A. millefolium* extracts, influencing, for example, their antioxidant activities [25,26]. One of the challenges connected with in vitro plant culture is a low level of bioactive compounds due to the lack of environmental stressors inducing their biosynthesis [27].

Decreased biosynthesis of secondary metabolites is a common phenomenon observed for plant in vitro cultures and the high accumulation of active compounds in in vitro plant material is rarely observed. Breeding without environmental stressors does not stimulate plants to produce various types of valuable secondary metabolites. This is confirmed by several studies identifying various factors affecting the phytochemical composition of plant material obtained from field and in vitro conditions [28,29,30]. Thus, the production and accumulation of biologically active compounds can be induced by introducing stress factors into the laboratory environment. For example, the process of shaking the medium leads to an increase in the content of phenolic acids (e.g., chlorogenic, rosemary or caffeic acids) in *Eryngium alpinum* L. in vitro cultures [31]. It was also described that the addition of biotic elicitors to the liquid medium increases the content of polyphenols in the tested plant material shoot cultures [32]. 

The biosynthesis of active compounds by plant in vitro cultures can also be regulated by solar radiation which may result in lower polyphenol production by in vitro cultures [28]. For this reason, the use of light of different intensities using darkness time during cultivation stimulates the growth and production of active compounds by in vitro cultures. The studies on in vitro plant cultures of *Verbena officinalis* L. comparing the effect of light on the production using media with a different supplementation of plant growth regulators (PGRs) showed interesting results. Increased synthesis of phenolic glycoside (verbazcoside) was observed in the dark in the MS medium with the addition of KIN, while in the case of the medium supplemented with BA, a higher content was observed in the light condition [33]. In addition, the color of light also significantly affects the accumulation of phenolic compounds in biomass from in vitro cultures. Blue light and white light showed the highest ability to stimulate the production of secondary metabolites of *Ruta graveolens* L. [34]. Moreover, using only blue light for the in vitro cultures of *Prunella vulgaris* L. showed a stimulation of the production of flavonoids and phenolic compounds [35]. All of the factors described above should be considered in the future experiments for increasing the production of bioactive metabolites by *A. millefolium* microshoot cultures.

### 2.3. Phytochemical Profiling

In addition to the total content of polyphenols, the extracts from AmL, AmH and AmIV were also analyzed for the content of particular metabolites using ultra-high-performance liquid chromatography coupled with ultra-high-resolution electrospray ionization quadrupole time-of-flight tandem mass spectrometry (UHPLC-HR-ESI-QTOF-MS/MS). The applied chromatographic conditions led to the separation of single constituents of the extracts on the column, and the mass spectrometer settings provided high-resolution mass measurement values that enabled the tentative identification of *A. millefolium* metabolites. The obtained mass chromatograms are presented in the Supplementary File (Figures S1–S3), whereas the list of identified components together with their percent relative abundance values is in Table 2 below.

Figure S1 of the 96% EtOH extracts from AmIV, AmL and AmH proves the presence of fatty acids being the major difference for AmL as compared to AmIV and AmH. Moreover, trihydroxy trimethoxyflavone at retention time 10.40 was also found to be the unique feature associated with AmL. Figure S2 of the 50% EtOH extracts from AmIV, AmL and AmH evidently highlights the differences observed among all three scrutinized extracts. The results confirm the high abundance of caffeoyl quinic acids in AmL extracts, whereas AmH extracts were rich in flavonoids and their glucosides. Figure S3 shows the H_2_O extracts and differentiating high abundance of caffeoyl quinic acids at RT 3.26 min and 6.58 min in two samples. The AmH showed high abundance of apigenin at RT 9.09 min. The score plots obtained from the principal component analysis (principal component 1 vs. 2) covering all extracts from AmIV, AmL and AmH are presented in Figure S4.

**Figure 2:**
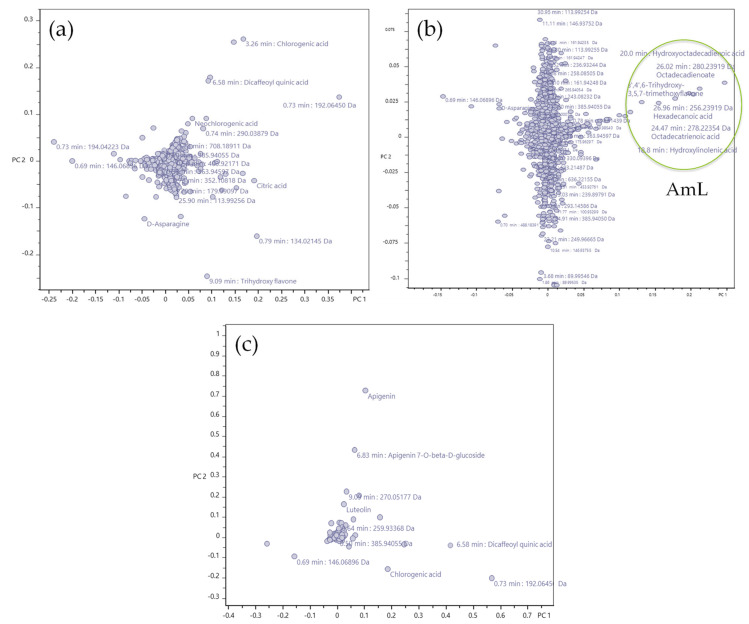
(**a**) Loadings plot of the principal component analysis (component 1 vs. component 2) covering all H_2_O extracts from AmIV, AmL and AmH. (**b**) Loadings plot of the principal component analysis (component 1 vs. component 2) covering all 96% EtOH extracts from AmIV, AmL and AmH. (**c**) Loadings plot of the principal component analysis (component 1 vs. component 2) covering all the 50% EtOH extracts from AmIV, AmL and AmH; the green circle indicates the presence of fatty acids, being the major difference for AmL extract as compared with AmIV and AmH.

Then, the loading plots for the principal component analysis covering all the extracts (Figure 2) were prepared and they showed that a group of certain signals has the greatest influence on the first two principal components. In H_2_O extracts, these were chlorogenic acid, dicaffeoylquinic acid, quinic acid, trihydroxy flavone (e.g., apigenin) and the hydrophilic region containing carbohydrates and acid fragments (Figure 2a). For the 96% EtOH extracts, these were 3′,4′,6-trihydroxy-3,5,7-trimethoxyflavone, hydroxylinolenic acid, hydroxyoctadecadienoic acid, octadecatrienoic acid, octadecadionate and hexadecanoic acid (Figure 2b). Finally, for the 50% EtOH extracts, these were apigenin, apigenin glucoside, chlorogenic acid, dicaffeoylquinic acid, quinic acid and trihydroxy flavone (e.g., apigenin) (Figure 2c).

### 2.4. Tyrosinase Inhibitory Activity

One of the most interesting cosmetic properties of *A. millefolium* extracts is their skin-lightening potential due to the presence of various tyrosinase inhibitors. Tyrosinase (EC.1.14.18.1, polyphenol oxidase) is a major enzyme of melanogenesis, catalyzing the first two rate-limiting reactions of melanin biosynthesis [36]. 

**Figure 3:**
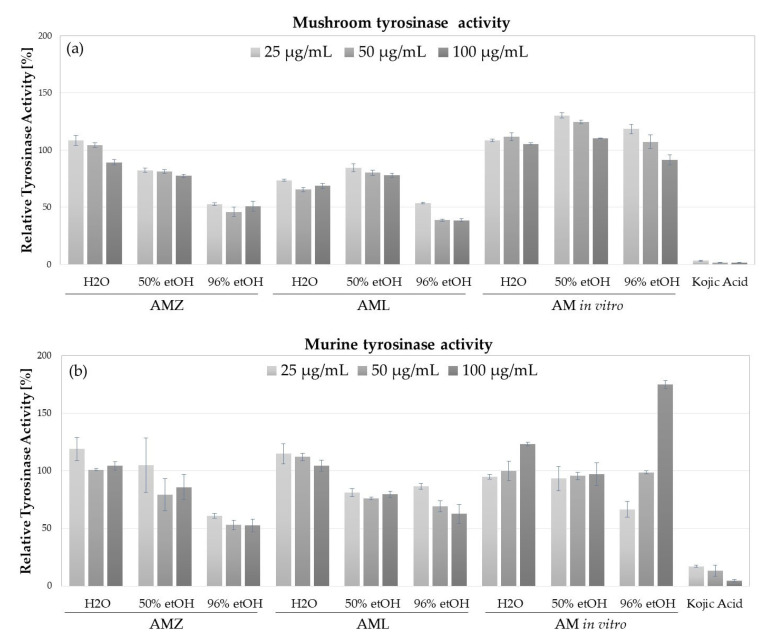
Inhibitory activity of *A. millefolium* extract obtained from the natural environment (AmH, AmL) and from in vitro microshoots culture (AmIV) on (**a**) mushroom tyrosinase and (**b**) murine tyrosinase, in comparison with kojic acid; graphs show mean values ± SD, *n = 3*.

Tyrosinase inhibitory potential was detected in analyzed AmH and AmL extracts (Figure 3). The highest inhibitory activity towards mushroom tyrosinase was detected for 96% EtOH AmH and AmL extracts, decreasing the activity of this enzyme by 50% at the highest tested concentration (100 µg/mL). Inhibition of mushroom tyrosinase was also found for 96% EtOH AmH and AmL extracts (15–23% inhibition) and H_2_O AmL extract (22% inhibition at 100 µg/mL) (Figure 3a). In respect of murine tyrosinase, contained in the lysate of B16F10 cells, the highest inhibitory potential was measured for 96% EtOH AmH extract (inhibitory rate from 39 to 48% at concentrations from 25 to 100 µg/mL). H_2_O and 96% EtOH AmL extracts also inhibited murine tyrosinase but to a lower level (38% inhibition at 100 µg/mL of 96% EtOH AmL) (Figure 3b). Interestingly, AmIV extracts prepared in different solvents did not inhibit mushroom or murine tyrosinases but, in fact, increased their activities. Lower tested concentrations (25 and 50 µg/mL) of 50% and 96% EtOH extracts increased mushroom tyrosinase activity by 10–30%. Additionally, 96% EtOH extract at 25 µg/mL inhibited murine tyrosinase by 34% and at 100 µg/mL increased the activity of the murine enzyme by 75%.

Tyrosinase inhibitory properties have been previously shown for ethyl acetate, methanolic, aqueous [8] hydroalcoholic [37] and hydroglycolic [5] extracts from this species. The studies involving activity-guided fractionation and identification of active compounds revealed that the derivatives of caffeolyquinic acid (e.g., 1,5-dicaffeolyquinic acid, 4,5-dicaffeolyquinic acid) present in *A. millefolium* extracts inhibit commercially available mushroom tyrosinase, whereas ferulic and caffeic acids are potent inhibitors of murine tyrosinase, obtained from murine melanoma cells B16F10 [37]. Although mushroom tyrosinase is the most commonly used in research on the skin-lightening potential of plant extracts, it has only 23% similarity in amino acid structure with human tyrosinase. The amino acid structure of murine tyrosinase is ca. 85% similar to the human enzyme [38]. Differences in inhibitory activity toward tyrosinases obtained from different sources were previously detected by our [37] and other research groups [39,40] and are considered as a significant factor influencing the in vivo activity of newly identified skin-lightening cosmetic ingredients.

Comparing the data on AmH, AmL and AmIV extracts’ tyrosinase inhibitory activity with their phytochemicals, it might be concluded that the significant mushroom tyrosinase inhibition of AmH and AmL extracts is caused by an abundance of chlorogenic acid, previously described as a potent mushroom tyrosinase inhibitor [37]. Other components of AmH and AmL extracts, including 3-caffeolyquinic acid (3-CQA), cynarin (1,3-DCQA) as well as flavonoids such as rutin, luteolin and its derivatives luteolin-7-glucoside and luteolin-7,3-di-*O*-glucoside, were also previously connected with mushroom tyrosinase inhibitory activity [8,41]. Murine tyrosinase inhibition detected for AmH and AmL extracts might also be partially caused by mentioned compounds or by other ingredients, for example, 3-O-feruloylquinic acid or 3-O-*p*-coumaroylquinic acid, as ferulic acid and quinic acid derivatives were also previously shown to inhibit tyrosinase from B16F10 cells [42,43]. 

Increased activity of murine tyrosinase caused by AmIV extracts might be explained by recent data published by Li and co-workers, who identified and purified three chlorinated sesquiterpene lactones (millefoliumin F, millefoliumin G, austricin) from *A. millefolium* which showed potent capacity to enhance the melanogenesis and tyrosinase activity in B16F10 murine melanoma cells [44]. In order to identify compounds responsible for the detected tyrosinase activating potential of AmIV extracts, further studies are required as the analytical method applied in this study did not allow for identification of sesquiterpene lactones.

## 3. Materials and Methods

### 3.1. Chemicals and Reagents

Ingredients for MS medium preparation and plant agar were bought from Duchefa Biochemie B.V, The Netherlands. PGRs—1-naphthaleneacetic acid (NAA), 6-benzyladenine (BA) and gibberellic acid (GA_3_) were bought from Merck Life Science Sp.z.o.o., an affiliate of Merck KGaA, Darmstadt, Germany. Mushroom tyrosinase from *Agaricus bisporus*, 3,4-dihydroxy-l-phenylalanine (L-DOPA), 2,2-diphenyl-1-picrylhydrazyl (DPPH) and standards of L-ascorbic acid, (3.3 g/L) and gallic acid were purchased from Sigma Aldrich (St. Louis, MO, USA). The purity of the reference compounds exceeded 95%. The HPLC-MS analyses were performed using the mass spectrometry purity solvents: water and acetonitrile (J.T. Baker, Phillipsburg, NJ, USA).

### 3.2. Plant Material and Extraction

#### 3.2.1. *Achillea millefolium* In Vitro Cultures

The material for the initiation of microshoot cultures of *Achillea millefolium* L. (yarrow) were seeds obtained from the collection of the Garden of Medicinal Plants, Jagiellonian University, Medical College, Faculty of Pharmacy, Cracow, Poland, in August 2021. The seeds were subjected to 5 min. sterilization with 0.1% HgCl_2_ (mercuric chloride) and then washed three times with sterile redistilled water and transferred to media (Figure 1a). After approximately two weeks, viable green microshoot cultures were obtained, which were transferred to a new medium every 3 weeks (Figure 1b). The experimental in vitro cultures were maintained on Murashige and Skoog medium [45] with 0.72% agar (*w*/*v*), 3% sucrose (*w*/*v*) and 10 mg/L vitamin B1. Plant growth regulators (PGRs) were added to the media: 1 mg/mL of cytokinin-6-benzylaminopurine (BA), 0.5 mg/mL of auxin-1-naphthaleneacetic acid (NAA) and 0.2 mg/mL of gibberellic acid (GA_3_). In vitro cultures were kept in special Magenta™ B-cap culture vessels (Sigma—Aldrich No. V8630, dimensions: 7 cm high, 6 cm diameter of the bottom of the vessel and 100 mL volume). Each culture vessel contained 30 mL of agar medium. In vitro cultures were cultivated at 25 ± 2 °C under continuous white artificial LED illumination with an intensity of 2.75 W/m^2^ (PPFD 40 μmol m^−2^ s^−1^). 

The multiplied biomass of agar microshoot cultures was used to establish experimental cultures. The weight of the inoculum was 0.5 g of fresh tissue. The growth time of the experimental cultures was 3 weeks (10 vessels per trial in 3 replicates). After the test period of growth, the in vitro culture biomasses and comparative material from the field conditions were frozen and then lyophilized using a LABCONCO lyophilizer (Kansas City, MI, USA). In order to obtain the value of biomass increments, their multiplicity was calculated using the parameter Gi—growth index [46]—which was calculated according to the following formula: Gi = (Dwn − Dw0)/Dwn) × 100, where Gi—growth factor biomass in time n (growth index); Dw0—inoculum dry mass; Dwn—dry mass after n time.

#### 3.2.2. *Achillea millefolium* Herbs and Leaves from the Field Condition

The plant material of herbs and leaves of *Achillea millefolium* was obtained from the collection of the Garden of Medicinal Plants, Jagiellonian University, Medical College, Faculty of Pharmacy, Cracow, Poland, in August 2021. Herbs and leaves were freeze-dried using a HarvestRight LLC lyophilizer (North Salt Lake, UT, USA). Voucher specimens of plant samples (AmL/2022/KGB and AmH/2022/KGB) were deposited at the Department of Cosmetology, University of Information Technology and Management in Rzeszów, Poland. 

#### 3.2.3. Extraction Procedure

Three types of extracts were obtained from AmH, AmL and AmIV plant material by mixing 250 mg of powdered plant with 5 mL of either 96% ethanol (96% EtOH extracts), 50% ethanol (50% EtOH extracts) or water (H_2_O extracts) and extracted using the digital ultrasonic water bath CD-482 (Hotair, Zawiercie, Poland) operating for 30 min at room temperature. The extracts (in triplicate) were evaporated to dryness using a Concentrator plus/Vacufuge^®^ plus (Eppendorf, Hamburg, Germany) at the temperature of 30 °C for 96% EtOH extracts and at 45 °C for 50% EtOH and H_2_O extracts. Dried residues were stored at −80 °C upon analysis.

### 3.3. Sample Preparation and UHPLC-UV-hr-qTOF-MS/MS Analysis

An amount of 20 mg of the dry extracts from *A. millefolium*, namely AmIV, AmL and AmH, was dissolved in their respective solvents, i.e., 96% EtOH extracts were dissolved in ethanol, 50% EtOH extracts in 50% ethanol and, similarly, H_2_O extracts were dissolved in water to a final concentration of 1 mg/mL. The prepared solutions were filtered through a PTFE syringe filter (0.45 microns) prior to the instrumental analysis.

The chromatographic fingerprinting of extracts was performed using the Maxis Impact Ultra High Resolution TOF-MS from Bruker Daltonics (Billerica, MA, USA) coupled to a Thermo Scientific Dionex UltiMate 3000. An Agilent RRHD Zorbax C8 (2.1 × 100 mm, 1.8 μm) column was used for the chromatographic separations. A gradient of acetonitrile (B) and 0.1 formic acid (FA) in water (A) was used as the mobile phase in the following linear steps (min/B%): 0/5%, 5/25%, 28/75%, 30/100%, 32/100%, 32.5/5%, 35/5%. The remaining method parameters were the following: the flow rate: 0.4 mL/min, the injection volume: 2 μL, column oven temperature: 40 °C. In the case of mass spectrometric measurements, an electrospray ionization in negative full scan and MS/MS mode was applied, in the mass range: 50–800 *m*/*z*, with the spectra rate collection: 4 Hz, nitrogen nebulizer gas pressure: 3 bar, drying gas flow rate: 12 L/min, end plate offset: 500 V, capillary voltage: 4500 V, dry temperature: 200 °C; funnel 1 RF and funnel 2 RF, 200 Vpp and 150 Vpp, respectively, CID energy: 0 eV, hexapole RF: 50 Vpp, quadruple ion energy: 5 eV. The UV spectra were recorded at 280 nm. Compass Data Analysis 4.2 software from Bruker^®^ (Billerica, MA, USA) was used for the interpretation of the mass-recorded signals. Each sample was measured in the form of technical duplicates.

### 3.4. Calibration, Data Cleansing, Peak Picking and Multivariate Analysis of the Recorded Spectral Data

The chemometric analysis of the recorded data that included the *m*/*z* calibration, the data cleaning, peak picking and the multivariate analyses was performed with Metaboscape 5.0 (Bruker daltonics, Billerica, MA, USA). The following program settings have been demarcated: (a) basis for peak picking: standard runs without acquisition of fragment spectra, (b) *m*/*z* calibration: using Na formate clusters in the retention time window 0.15 min–0.3 min, (c) *m*/*z* range for peak picking: 50–800 *m*/*z*, according to the measurements, (d) retention time window for peak picking: 0.5 min–31.0 min, (e) minimum number of features for extraction: 1/33, (f) minimum number of features for the result: 1/33, (g) minimum cluster length for extraction: 6, (h) minimum seed cluster length: 6, (i) sample grouping filter: 25, (j) minimum intensity: 2000, (k) algorithm used: T-ReX 3D Default Processing, (l) Ion Deconvolution: T-ReX Metabolomics Negative Ions, (m) EIC correlation: 0.9, (n) primary ion: [M-H]^−^, (o) common ion: [M-H-H_2_O]^−^, (p) seed ions: [M + HCOO]^−^. 

For multivariate analysis, the following parameters for principle components analysis (PCA) were selected: scaling algorithm—Pareto, cross validation mode 10 percent, minimal explained variance 98.0. Due to the determined model, quality comparisons of the two main components were made.

### 3.5. Total Polyphenolic Content

Total polyphenolic content was measured according to the spectrophotometric method described by Fukumoto and Mazza [47] using the gallic acid standard curve. The content of total phenolics was calculated as gallic acid equivalents (GAEs) in mg per g of dried extract.

### 3.6. DPPH Scavenging Activity Assay

Antioxidant activity of AmL, AmH and AmIV extracts was analyzed using DPPH radical scavenging assays as described by Matejic et al. [48]. Briefly, 100 μL of diluted extracts (500–0.49 µg/mL) or L-ascorbic acid (1000–0.98 µg/mL) was mixed with 100 μL DPPH working solution (25 mM in 99.9% methanol; A540 ≈ 1). An amount of 100 μL of methanol solvent mixed with 100 μL DPPH was used as a control sample (100% DPPH radical activity). Following 10 min incubation at room temperature in darkness, the absorbance of the samples was measured at λ = 540 nm using a microplate reader (FilterMax F5 Molecular Devices, San Jose, CA, USA). The percentage of DPPH radical scavenging was calculated based on the following equation:

% of DPPH radical scavenging = [1−(Abs(S)/Abs(C))] × 100

where Abs(S)—the absorbance of the extract, Abs(C)—the absorbance of the control sample. The IC_50_ value was defined as the concentration of dried extract in µg/mL that is required to scavenge 50% of the radical activity and calculated using GraphPad Prism 9.0 software (San Diego, CA, USA).

### 3.7. Mushroom and Murine Tyrosinase Inhibitory Assay

The tyrosinase inhibitory activity of AmL, AmH and AmIV extracts was compared using commercially available mushroom tyrosinase and murine tyrosinase in the lysate of B16F10 murine melanoma cells (ATCC CRL-6475; LGC Standards, Łomianki, Poland), obtained as previously described [36]. The mushroom tyrosinase activity assay was performed based on the method by Uchida et al. [49]. Briefly, 120 μL phosphate buffer (100 mM, pH = 6.8) was mixed with 20 μL of diluted extracts (1, 0.5 and 0.25 mg/mL) and 20 μL of mushroom tyrosinase (500 U/mL) and pre-incubated at room temperature for 10 min. Following the addition of 40 μL L-DOPA (4 mM), the samples were incubated for another 20 min at RT. The activity of murine tyrosinase was measured by mixing the volume of B16F10 lysate containing 20 µg protein with 20 µL of diluted extract (1, 0.5 and 0.25 mg/mL), 40 µL 4 mM L-DOPA and 100 mM phosphate buffer pH 6.8 (up to 200 µL). The reaction was carried out for 4 h at 37 °C. Control samples (100% tyrosinase activity) for both assays contained the appropriate volume of the solvent instead of the extract. In both assays, the dopachrome formation was measured spectrophotometrically at λ = 450 nm using a FilterMax F5 microplate reader (FilterMax F5 Molecular Devices, USA). The obtained values were corrected by the absorbance value of the extracts without mushroom or murine tyrosinase and L-DOPA. Each sample was analyzed in 3 independent repetitions. Kojic acid at comparable concentrations was used as a known tyrosinase inhibitor control.

## 4. Conclusions

The data described in this paper present the conditions of a simple method for the establishment and cultivation of *A. millefolium* microshoot in vitro cultures, which should be optimized in the future in order to modify the phytochemical composition of the plant. The phytochemical content of plant material obtained from in vitro conditions was significantly different from the plants growing in the field. The total content of polyphenols was about 7–10 times lower in AmIV than in AmL and AmH extracts, not exceeding 0.25 mg GAE/g of dried extract. A low content of polyphenols was most likely responsible for the lack of antioxidant activity and tyrosinase inhibitory properties that were detected in AmH and AmL but not in AmIV extracts. AmIV extracts were shown to increase the activity of mushroom tyrosinase and tyrosinase present in B16F10 murine melanoma cells.

To summarize, the presented data clearly indicate the need of further experimental studies aiming to identify the factor regulating biosynthesis and accumulation of polyphenolic compounds in *A. millefolium* in vitro cultures. The identified differences in the content of individual metabolites between the plant material from in vitro and from the field indicate which precursor metabolites can be supplemented to *A. millefolium* in vitro cultures to increase the content of biologically important compounds. Further studies involving various compositions of culture medium and different types of in vitro cultures (agitated cultures, the usage of bioreactors) will help to optimize *A. millefolium* microshoot cultures as valuable raw materials for the cosmetic industry.

## Figures and Tables

**Table 1:** The content of polyphenolic compounds and DPPH scavenging activity of AmH, AmL and AmIV extracts; mean ± SD, *n = 3*.

		Total Polyphenols (mg GAE/g Dried Extract)	Dpph Scavenging (EC_50_; µg/mL)
AmH	H_2_O	0.87 ± 0.01	26.12 ± 0.86
	50% EtOH	1.71 ± 0.04	8.22 ± 2.32
	96% EtOH	0.55 ± 0.01	20.49 ± 3.56
AmL	H_2_O	1.51 ± 0.04	24.93 ± 0.69
	50% EtOH	2.63 ± 0.11	5.10 ± 0,04
	96% EtOH	0.46 ± 0.01	38.89 ± 3.25
AmIV	H_2_O	0.13 ± 0.01	>500
	50% EtOH	0.24 ± 0.06	400.05 ± 45.47
	96% EtOH	0.06 ± 0.01	>500
L-ascorbic acid		-	1.47 ± 0.29

**Table 2:** The list of compounds tentatively identified in the studied extracts as determined through the data base search of the tandem mass spectra from the compounds (NIST 2020 MSMS Spectral Library incorporated to Metaboscape^®^ 5.0) together with their relative abundance (Tr—0–5; + 6–30%; ++ 31–60; +++ 61–100%).

No.	RT [min]	*m*/*z* Meas.	M Meas.	Ions	Name	Compound Class	AmL	AmH	AmIV
1	0.69	131.04573	132.05258	[M-H]^−^, [M-H-H_2_O]^−^	D-Asparagine	Amino acids	++	+++	+++
2	0.84	191.01948	192.02677	[M-H]^−^, [M-H-H_2_O]^−^	Citric acid	AHA- alpha hydroxy acid	+++	+++	+++
3	1.28	164.07129	165.07857	[M-H]^−^	3-Phenyl-β-alanine	Amino acids	Tr	Tr	Tr
4	1.41	315.07089	316.07808	[M-H]^−^	Gentisic acid 5-O-glucoside	Phenolic acid	+++	+++	+
5	1.84	353.08612	354.09339	[M-H]^−^	Neochlorogenic acid	Phenolic acid	+++	++	Tr
6	2	203.08181	204.08908	[M-H]^−^	L-Tryptophan	Amino acids	+++	+++	+
7	2.6	137.02531	138.03258	[M-H]^−^	3,4-Dihydroxybenzaldehyde	Phenolic aldehyde	Tr	Tr	Tr
8	3.27	353.08673	354.09401	[M-H]^−^	Chlorogenic acid	Phenolic acid	+++	++	Tr
9	3.68	353.08617	354.09345	[M-H]^−^	(1R,3R,4S,5S)-4-(((2E)-3-(3,4-Dihydroxyphenyl)prop-2-enoyl)oxy)- -1,3,5-trihydroxycyclohexanecarboxylic acid	Phenolic acid	Tr	Tr	Tr
10	4.55	353.08609	354.09337	[M-H]^−^	Chlorogenic acid	Phenolic acid	+++	+	Tr
11	4.74	311.07578	312.08306	[M-H]^−^	4-[(2E)-3-(3,4-Dihydroxyphenyl)prop-2-enoyl]oxy-2,3-dihydroxy- -2-methylbutanoic acid	Phenolic acid	Tr	Tr	Tr
12	4.78	515.11701	516.12428	[M-H]^−^	Cynarin	Phenolic acid	+++	+	Tr
13	5.07	367.10169	368.10897	[M-H]^−^	3-O-Feruloylquinic acid	Phenolic acid	+++	+++	Tr
14	5.09	609.14408	610.15136	[M-H]^−^	Luteolin-7,3′-di-O-glucoside	Flavonoid glucosides	+++	+++	Tr
15	5.23	371.09659	372.10387	[M-H]^−^	3-(Benzoyloxy)-2-hydroxypropyl.beta.-D-glucopyranosiduronic acid	Phenolic acid	Tr	Tr	Tr
16	5.29	563.13792	564.14519	[M-H]^−^	Schaftoside	Flavonoids	+	+++	Tr
17	5.35	463.08594	464.09322	[M-H]^−^	Hyperin	Flavonoid	+++	+++	Tr
18	5.38	221.04479	222.05207	[M-H]^−^	Fraxidin	Coumarin	Tr	+++	+++
19	5.43	337.09133	338.09861	[M-H]^−^	3-O-*p*-Coumaroylquinic acid	Phenolic acid	+++	+++	Tr
20	5.46	595.12842	596.13568	[M-H]^−^	Peltatoside	Flavonoids	+++	+	Tr
21	5.82	609.14383	610.15111	[M-H]^−^	Rutin	Flavonoids	+++	+++	Tr
22	6.13	447.09125	448.09853	[M-H]^−^	Luteolin 7-glucoside	Flavonoid glucosides	+++	+++	Tr
23	6.66	577.15398	578.16126	[M-H]^−^	Spherobioside	Flavonoids	+++	+++	Tr
24	6.74	447.09085	448.09813	[M-H]^−^	Juncein	Flavonoid glucosides	+++	+++	Tr
25	6.83	431.0972	432.10632	[M-H]^−^	Dienin 7-O-beta-D-glucoside	Flavonoid glucosides	Tr	Tr	Tr
26	6.88	445.07551	446.08279	[M-H]^−^	Apigenin 7-glucuronide	Flavonoid glucosides	+++	+++	Tr
27	6.92	607.16497	608.17225	[M-H]^−^	Chrysoeriol 7-neohesperidoside	Flavonoids	+++	++	Tr
28	7.05	475.08592	476.09319	[M-H]^−^	Hispidulin 7-glucuronide	Flavonoids	+++	+++	Tr
29	7.63	269.04456	270.05183	[M-H]^−^	Apigenin	Flavonoids	+++	+++	Tr
30	7.73	417.08072	418.08799	[M-H]^−^	Juglanin	Flavonoids	+++	++	Tr
31	8.06	285.03923	286.04651	[M-H]^−^	Luteolin	Flavonoids	+++	+++	Tr
32	8.28	591.16978	592.17734	[M-H]^−^, [M + HCOO]^−^	5-Hydroxy-3-(4-methoxyphenyl)-4-oxo-4H-chromen-7-yl 6-O-(6-deoxyhexopyranosyl)hexopyranoside	Flavonoid glucosides	Tr	Tr	Tr
33	8.56	207.06537	208.07265	[M-H]^−^	Ethyl *trans*-caffeate	Phenolic compound	+++	+++	+
34	9.09	269.04456	270.05183	[M-H]^−^	Trihydroxyflavone or Apigenin	Flavonoids	+++	+++	Tr
35	9.34	329.06492	330.07224	[M-H]^−^	3,4′-Dimethoxy-5,7,3′-trihydroxyflavone	Flavonoids	Tr	Tr	Tr
36	9.35	299.05464	300.06191	[M-H]^−^	Luteolin 7-methyl ether	Flavonoids	+++	+++	Tr
37	9.62	329.06515	330.07243	[M-H]^−^	Cirsiliol	Flavonoids	+++	+++	Tr
38	10.4	359.07595	360.08322	[M-H]^−^	3′,4′,6-Trihydroxy-3,5,7-trimethoxyflavone	Flavonoids	+++	Tr	Tr
39	11.65	329.23187	330.23915	[M-H]^−^	(9Z)-5,8,11-Trihydroxyoctadec-9-enoic acid	Fatty acid	++	+++	+++
40	12.17	283.05974	284.06702	[M-H]^−^	Genkwanin	Flavonoids	+++	+++	Tr
41	12.2	373.09123	374.09854	[M-H]^−^	Pulicarin	Flavonoids	+++	+	Tr
42	12.48	313.07013	314.07741	[M-H]^−^	Pectolinarigenin	Flavonoids	+++	+++	Tr
43	13.69	343.08068	344.08796	[M-H]^−^	5,3′-Dihydroxy-6,7,4′-trimethoxyflavone	Flavonoids	Tr	Tr	Tr
44	18.81	2293.2041	294.21944	[M-H]^−^	Hydroxylinolenic acid	Fatty acid	Tr	Tr	Tr
45	19.93	221.15373	222.16132	[M-H]^−^	3,6-Ditert-butyl-1,2-benzenediol	Phenolic compound	++	+++	+++
46	20	295.21923	296.23522	[M-H]^−^	Hydroxyoctadecadienoic acid	Fatty acid	++	+++	+++
47	20.24	452.27606	453.28333	[M-H]^−^	1-Palmitoyl-2-hydroxy-sn-glycero-3-phosphoethanolamine	Phospholipid	Tr	Tr	Tr
48	20.95	265.14661	266.15389	[M-H]^−^	Laurylsulfuric acid	Organic acid	+	+++	+
49	23.7	271.22659	272.23386	[M-H]^−^	2-Hydroxypalmitic acid	Fatty acid	+++	++	++
50	24.47	277.21072	278.22335	[M-H]^−^	Octadecatrienoic acid	Fatty acid	Tr	Tr	Tr
51	25.17	483.27031	484.27759	[M-H]^−^	1-Palmitoyl-2-hydroxy-sn-glycero-3-phospho-(1′-rac-glycerol)	Phospholipid	Tr	Tr	Tr
52	26.02	279.22581	280.239	[M-H]^−^	Octadecahenate	Organic acid	Tr	Tr	Tr
53	26.96	255.22641	256.239	[M-H]^−^	Hexadecanoic acid	Fatty acid	Tr	Tr	Tr
54	27.2	339.23123	340.2385	[M-H]^−^	2,2′-Methylene-bis(6-tert-butyl-4 methylphenol)	Phenolic compound	Tr	Tr	Tr

## Data Availability

All data are included in the manuscript and Supplementary File.

## Supplementary Materials

The following supporting information can be downloaded at https://www.mdpi.com/article/10.3390/molecules28124791/s1, Figure S1: Listed base peak chromatograms for ethanol extracts from AmIV, AmL and AmZ. Chromatograms highlight the unique features in AmL extracts.; Figure S2. Listed base peak chromatograms for 50% ethanol extracts from AmIV, AmL and AmZ in the negative ionization mode. Chromatograms highlight the high abundance of caffeoyl-quinic acids in AmL extracts, while AmZ extracts were found rich in flavonoids and their glucosides; Figure S3. Listed base peak chromatograms for water extracts from AmIV, AmL and AmZ (AmL unveils high amount of caffeoyl-quinic acids) recorded in the negative ionization mode; Figure S4. (a) Scores plot of principal component analysis covering all the water extracts from AmIV, AmL and AmZ. (b) Scores plot of principal component analysis encompassing all the ethanol extracts from AmIV, AmL and AmZ. (c) Scores plot of principal component analysis covering all the 50% ethanol extracts from AmIV, AmL and AmZ.

## Sample Availability

Samples of the AmL, AmH and AmIV extracts and dried *A. millefolium* herbs and leaves are available from the authors.
