# Supplementary material for: Phytochemical Profiling, Antioxidant and Tyrosinase Regulatory Activities of Extracts from Herb, Leaf and In Vitro Culture of Achillea millefolium (Yarrow)
Source: Molecules. 2023 Jun 15;28(12):4791. doi: 10.3390/molecules28124791 (PMC10305548; doi:10.3390/molecules28124791)
Supplement: Supplementary file 1 [file molecules-28-04791-s001.zip › molecules-2405682-supplementary.pdf]

Phytochemical profiling, antioxidant and tyrosinase regulatory activities of extracts from herb, leaf and in vitro culture of *Achillea millefolium* (yarrow)

Karolina Czech, Katarzyna Gaweł-Bęben, Agnieszka Szopa, Wirginia Kukuła-Koch, Thomas Jakschitz, Günther Bonn, Faryal Rubaba, Hira Ijaz, Shah Hussain, Paweł Kubica, Halina Ekiert and Kazimierz Głowniak

Supplementary materials

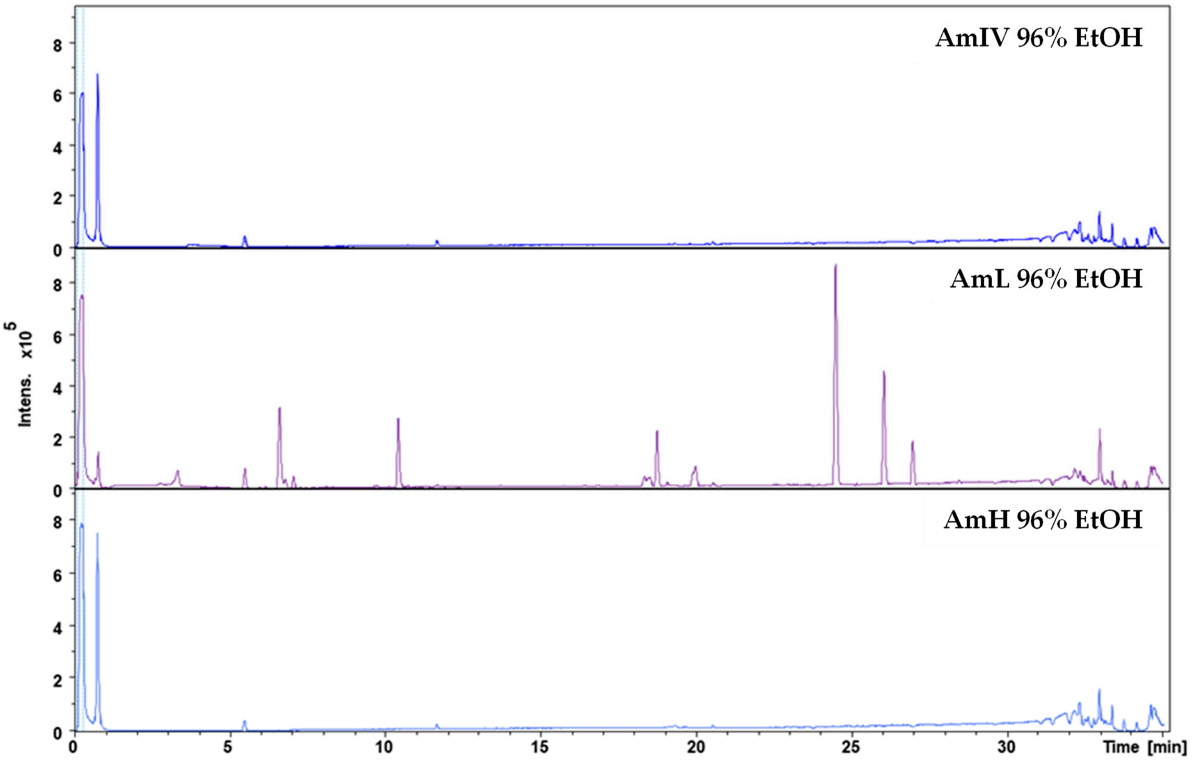

Figure S1. Listed base peak chromatograms for ethanol extracts from AmIV, AmL and AmZ. Chromatograms highlight the unique features in AmL extracts.

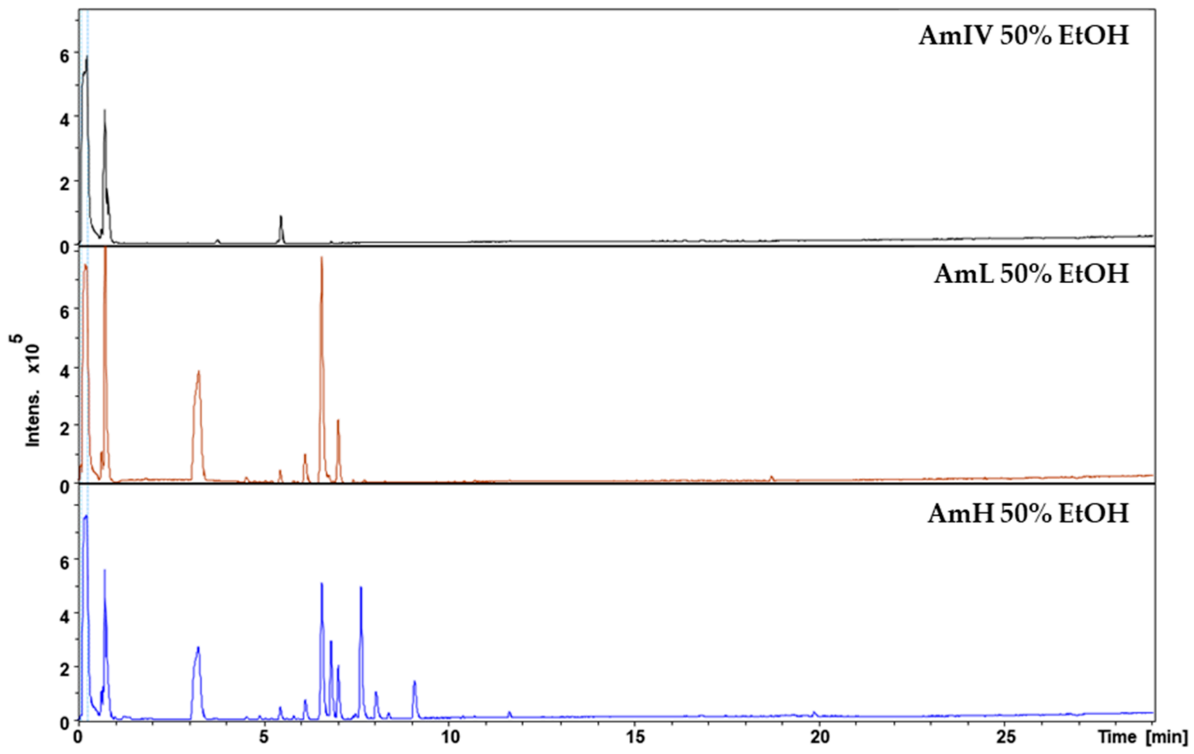

Figure S2. Listed base peak chromatograms for 50% ethanol extracts from AmIV, AmL and AmZ in the negative ionization mode. Chromatograms highlight the high abundance of caffeoyl-quinic acids in AmL extracts, while AmZ extracts were found rich in flavonoids and their glucosides.

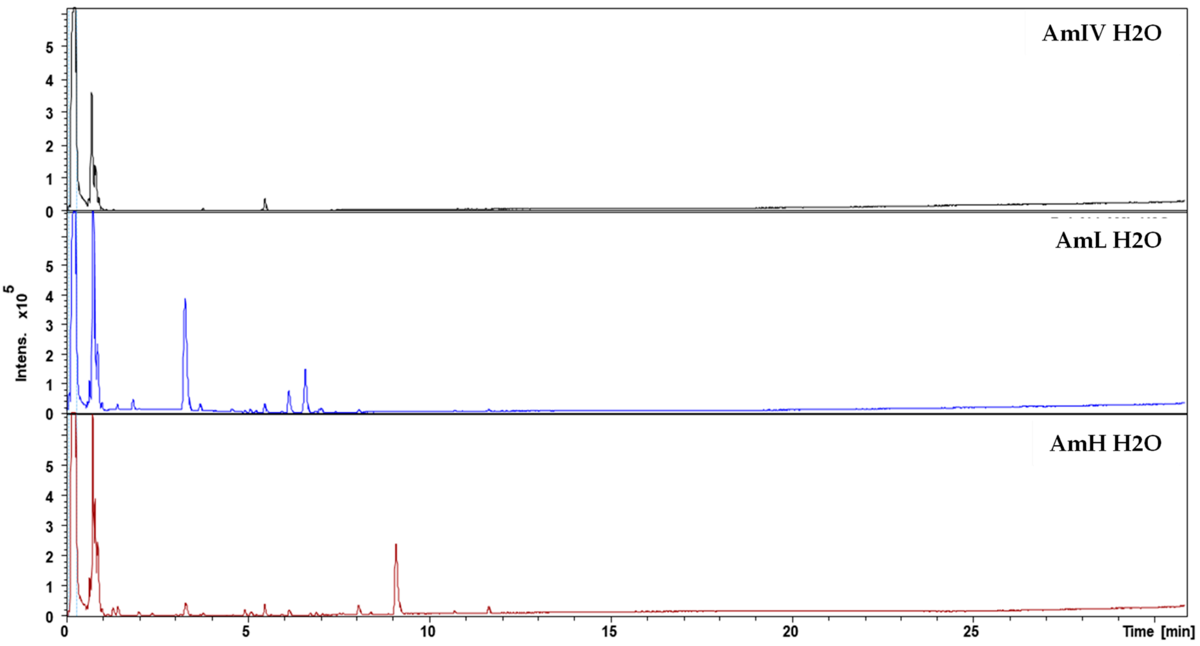

Figure S3. Listed base peak chromatograms for water extracts from AmIV, AmL and AmZ (AmL unveils high amount of caffeoyl-quinic acids) recorded I the negative ionization mode.

The score plots obtained from the principal component analysis (principal component 1 versus 2) covering all water extracts from AM, AML and AMZ are presented in Figure S4 (A), 50% ethanol extracts (C). The statistics related to the principal component analysis covering all water extracts were: (component 2, R2: 0.680, Q2 0.624, component 3, R2: 0.765, Q2 0.676, component 4, R2: 0.934, Q2 0.378, component 5, R2: 1.000, Q2 0.378). Concerning 50 % ethanol extracts: component 2 R2: 0.405, Q2 0.330, component 3 R2: 0.917, Q2 0.153, component 4 R2: 0.956, Q2 0.149 component 5, R2: 1.000, Q2 0.149. Int he case of ethanol extracts (B) the Statistics connected to principal component analysis covering all the extracts were: (component 2 R2: 0.850, Q2 0.672, component 3 R2: 0.868, Q2 0.680, component 4 R2: 0.880, Q2 0.682, component 5 R2 1.000 Q2 0.682.

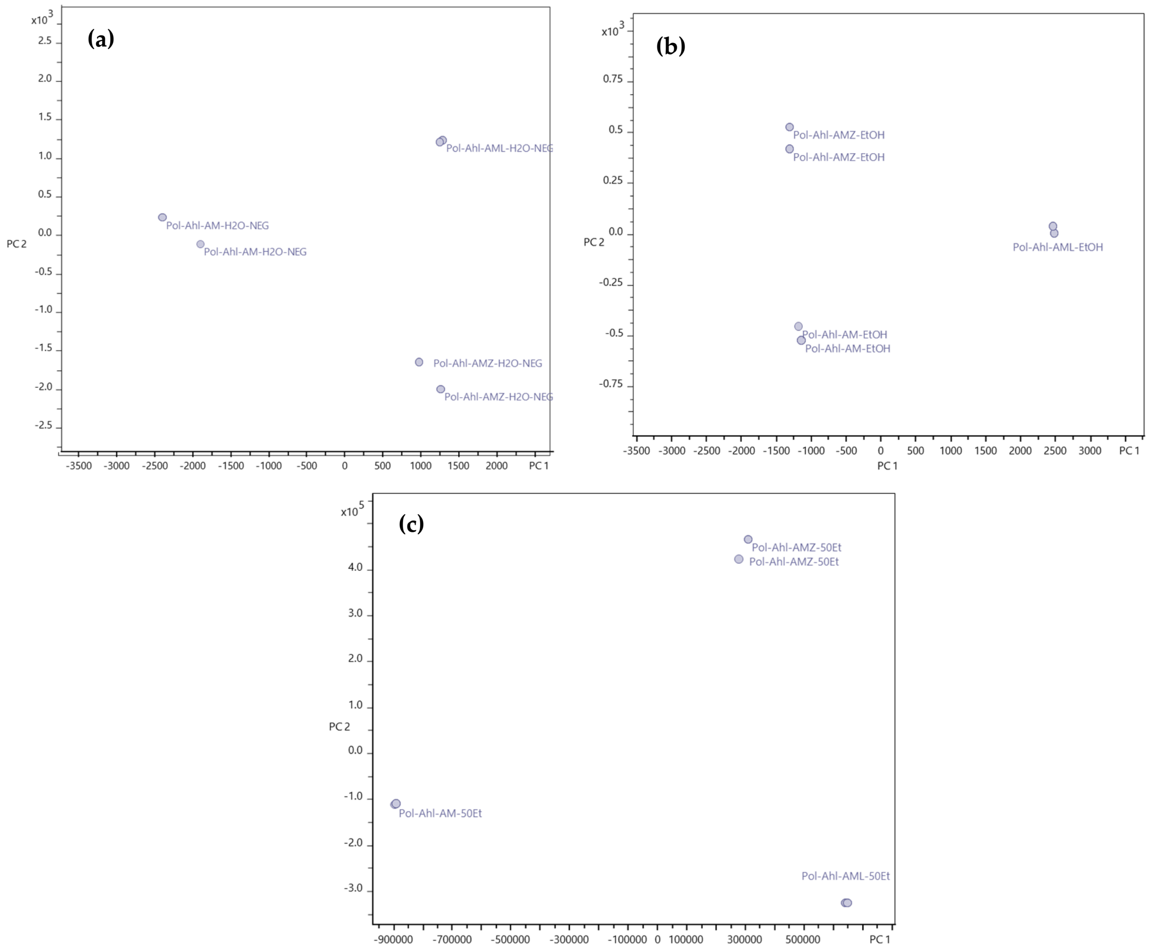

Figure S4. (a) Scores plot of principal component analysis covering all the water extracts from AmIV, AmL and AmZ (b) Scores plot of principal component analysis encompassing all the ethanol extracts from AmIV, AmL and AmZ (c) Scores plot of principal component analysis covering all the 50% ethanol extracts from AmIV, AmL and AmZ
